# Genome-wide association study of agronomical and root-related traits in spring barley collection grown under field conditions

**DOI:** 10.3389/fpls.2023.1077631

**Published:** 2023-01-24

**Authors:** Piotr Ogrodowicz, Krzysztof Mikołajczak, Michał Kempa, Monika Mokrzycka, Paweł Krajewski, Anetta Kuczyńska

**Affiliations:** Institute of Plant Genetics, Polish Academy of Sciences, Poznan, Poland

**Keywords:** barley, genome-wide association studies, drought, roots architecture, gene annotations

## Abstract

The root system is a key component for plant survival and productivity. In particular, under stress conditions, developing plants with a better root architecture can ensure productivity. The objectives of this study were to investigate the phenotypic variation of selected root- and yield-related traits in a diverse panel of spring barley genotypes. By performing a genome-wide association study (GWAS), we identified several associations underlying the variations occurring in root- and yield-related traits in response to natural variations in soil moisture. Here, we report the results of the GWAS based on both individual single-nucleotide polymorphism markers and linkage disequilibrium (LD) blocks of markers for 11 phenotypic traits related to plant morphology, grain quality, and root system in a group of spring barley accessions grown under field conditions. We also evaluated the root structure of these accessions by using a nondestructive method based on electrical capacitance. The results showed the importance of two LD-based blocks on chromosomes 2H and 7H in the expression of root architecture and yield-related traits. Our results revealed the importance of the region on the short arm of chromosome 2H in the expression of root- and yield-related traits. This study emphasized the pleiotropic effect of this region with respect to heading time and other important agronomic traits, including root architecture. Furthermore, this investigation provides new insights into the roles played by root traits in the yield performance of barley plants grown under natural conditions with daily variations in soil moisture content.

## Introduction

Nowadays, barley (*Hordeum vulgare* L.) is the fourth most important crop in terms of grain production (FAO, 2021). Because of the synteny of grass genomes, it is considered a model crop, being diploid with a genome size of 5.3 Gbp (Hori et al., 2003). Previous studies have shown that barley production is severely affected by abiotic stresses such as drought and heat (Sallam et al., 2019). In order to cope with the effects of climate change, breeders make attempts to improve the adaptability and stability of yield performance of new varieties of barley. As a result, many breeding programs now focus on the adaptation of barley varieties to climate change. For any crop, the root structure is a promising target to increase yield, especially under unfavorable growth conditions. Incorporating genetic information on root traits and root architecture into breeding practices has been reported to possibly improve resource efficiency or stress tolerance.

For the uptake of water and nutrients from the deep zone of the soil, plants require roots with a larger number of root tips (Osmont et al., 2007). From the perspective of breeding, it would be desirable to have genotypes with a larger root volume and a higher dry weight and other related parameters so that they can extract a high amount of water and continue to grow under drought stress (Danakumara et al., 2021).

Nondestructive evaluation of the root growth of plants remains a challenge in research on the root system. Plant roots, in particular, the dynamics of root growth, have not been studied as intensively as shoots, mainly because of physical constraints. Thus far, several methods have been used to study root growth, including core sampling; ingrowth cores; excavation of the root system; X-ray imaging; magnetic resonance imaging; and application of monoliths, ground-penetrating radar, isotopes, rhizotrons, minirhizotrons, and electrical capacitance (Chloupek, 1972; Prokushkin, 1982; van Beem et al., 1998; Hruska et al., 1999). Electrical capacitance is used to study root growth on the basis of the assumption that the capacitance of the root–soil system changes with an increase in the contact area between roots and soil following plant growth (Chloupek, 1977; Dalton, 1995). Dietrich et al. (2013) showed that it is difficult to evaluate root system morphology based on electrical capacitance values. However, the primary advantage of this method is the possibility to measure the electrical capacitance of hundreds of plants per day and to repeat the measurements at different phenological stages. Because dying membranes lose their electrical capacitance, the measured capacitance reflects not only the size of the root system but also the vitality of the membranes. In recent years, the size of the root system, which is measured using the electrical capacitance method, has been used as a criterion to select drought-resistant genotypes in crops such as spring barley (Chloupek et al., 2010; Svačina et al., 2014) and winter wheat (Abdel-Ghani et al., 2013).

economic traits of crops are complex quantitative ones (e.g., yield, quality, earliness, and responses to biotic and abiotic stresses) and have been the focus of interest of plant breeders and researchers for decades. As these traits have been explored only to a limited extent because of their low heritability and environmental sensitivity, they have eventually become the target of traditional breeding approaches; however, expensive and labor-intensive phenotyping procedures are required to successfully explore these quantitative traits (Bhat et al., 2015). Genomic approaches are particularly useful to analyze complex traits as they are usually multigenic in nature and are significantly influenced by the environment. Next-generation sequencing (NGS) has significantly reduced the cost and time needed to sequence and identify single nucleotide polymorphisms (SNPs). Thus, sequence-based genotyping has been developed for the application of NGS in plant breeding and genomic studies. The development of the genotyping-by-sequencing (GBS) approach has led to the introduction of SNPs that are suitable and affordable for genomic selection in both model and nonmodel plant species (Poland et al., 2012). GBS is widely used for genetic mapping of economically important crops (Elshire et al., 2011), including barley (Liu et al., 2014; Yao et al., 2018).

Recently, genome-wide association study (GWAS) has been recognized as a powerful approach to identify genes that regulate complex traits in crops (Famoso et al., 2011; Huang et al., 2012; Morris et al., 2013; Gali et al., 2019; Muhammad et al., 2021). GWAS allows to identify genes that influence natural variations in quantitative traits through the association of markers’ polymorphism with phenotypic variation within diverse panels (Xiao et al., 2017); thus, insights into the genetic architecture of important and yield- and root-related traits can be obtained. This approach has recently become popular for genetic mapping of quantitative traits and for studying natural variations. As reported earlier, linkage disequilibrium (LD) blocks that combine two or more SNPs are more informative than single biallelic SNPs (Rochat et al., 2007). Lorenz et al. (2010) defined barley haplotypes by using different analytical methods and confirmed that the distribution of quantitative trait locus (QTL) alleles in nature does not match that of marker variants; thus, haplotype information could allow to capture associations that would elude individual SNPs. Recent GWASs in wheat (Sehgal et al., 2020) and other crops (Ogawa et al., 2018; Siekmann et al., 2021) have shown that a haplotype-based approach improves prediction accuracy as compared to an individual SNP approach. It has also been suggested that compared to individual markers, haplotype-based GWAS better represents the genetic architecture of complex traits, as this approach can capture epistatic interactions between SNPs at a locus (Bardel et al., 2005) and can more accurately predict whether an allelic series exists at a locus (Hamblin et al., 2011).

The objectives of the present study were as follows: (i) to investigate the phenotypic variations of selected root- and yield-related traits in a diverse panel of spring barley genotypes, (ii) to reveal their genetic architecture by using LD-based GWAS, and (iii) to analyze the potential association between traits linked to root- and yield-related properties of the studied accessions. To assess root architecture in the field, the electrical capacitance characteristics of barley roots and manual root traits were evaluated.

## Materials and methods

### Plant material and field trials

A total of 149 spring barley genotypes (accession panel) were used in phenotypic and genotypic analyses. The studied accessions were obtained (with few exceptions) from the Polish Breeding Companies. A majority of the barley accessions (85) were registered as cultivars, and 54 accessions were classified as breeding lines. To increase the diversity of the panel, four RILs (seed collection of Institute of Plant Genetics Polish Academy of Sciences – IPG PAS) and eight BC lines (six from IPG PAS collection and two from NordGen collection) were also included. We focused mainly on two-row spring barley accessions from breeding companies’ collections to reduce the confounding effects of population structure in terms of origin, growth, and row types (Comadran et al., 2012; Darrier et al., 2019). The complete list of the accessions used in this study is given in **Supplementary Table 1**.

The study was conducted over three growing seasons (2017, 2018, 2020) on the experimental fields located at IPG PAS in Poznań, Poland (52°24′30″N 16°56′03″E). All trials were performed in three replications, in randomized blocks, and under standard fertilization and cultivation conditions. The experimentalplots consisted of five rows of 150-cm length that were spaced 20 cm apart. Seeds were planted within each row at 15 cm from each other. In each of the replications, there were 10 individual plants. Before sowing, the seeds were treated with the systemic fungicide Funaben. During the experiment, spraying was carried out as needed to protect the plants against diseases or pests required. Standard agricultural practices of barley production were followed for field management, including irrigation, fertilization, weed control, and pestmanagement. More information in terms of soil fertilization can be found in **Supplementary Table 2**.

As shownin **Table 1**, weather data included monthly (March–August) average values from 2017, 2018 and 2020, which were obtained from the Institute of Meteorology and Water Management—National Research Institute database(IMWM–NRI). In addition, soil moisture was evaluated using a handheld moisture meter TDR (Suchorab et al., 2014) attached to an FOM/mts field TDR probe with a 150-mm rod length (E-Test, Poland). The measurements were taken in six randomly selected spots (with three replicates) in the experimental field at three different plant phenological stages (T1, tillering—BBCH 23; T2, stem elongation—BBCH 33; and T3, heading—BBCH 56), as shown in **Table 2**.

**Table 1:** Average monthly weather conditions recorded for the growing period (March–August) in 2017, 2018 and 2020.

Month/year	Air temperature (°C)			Monthly precipitation (mm)			Air humidity (%)			No. of days with rainfall		
	2017	2018	2020	2017	2018	2020	2017	2018	2020	2017	2018	2020
March	6.7	1.0	5.2	37.5	29.4	29.0	71.3	72.1	65.7	18	16	15
April	7.7	13.4	9.8	33.9	28.1	2.6	71.5	64.7	50.2	18	19	6
May	14.2	17.9	12.0	32.9	16.6	47.5	67.7	55.9	63.1	14	11	16
June	18.1	19.4	18.5	87.0	22.8	51.3	66.5	58.1	70.8	18	12	17
July	18.6	20.9	19.3	127.1	81.3	65.3	71.9	63.7	61.6	24	13	15
August	19.3	21.7	21.0	97.0	9.8	60.9	71.4	57.2	63.4	12	11	15

**Table 2:** Soil moisture (volumetric water content) recorded (mean values) at six randomly chosen spots in the field.

No. of field sites	Soil moisture (%)								
	2017			2018			2020		
	T1	T2	T3	T1	T2	T3	T1	T2	T3
1	10.3	12.6	19.1	13.7	7.7	2.6	8.5	13.1	9.1
2	8.1	11.8	16.2	14.1	10.8	2.2	7.4	11.2	11.0
3	8.5	9.8	14.4	13.8	9.2	2.6	7.9	11.8	10.5
4	7.4	8.5	10.8	11.5	7.0	6.0	6.5	8.1	11.7
5	15.0	11.8	9.5	11.9	8.6	4.0	6.9	10.0	11.1
6	9.5	10.8	15.3	13.8	6.8	2.6	7.5	14.2	10.7
**Total**	**9.5**	**10.8**	**13.8**	**13.1**	**8.2**	**3.1**	**7.4**	**11.2**	**10.7**

The soil moisture conditions were measured by the hand-held device - FOM/mts instrument.

### Trait evaluation

Once plants reached full maturity, they were harvested, preserved, and then subjected to biometric analysis in which the following agronomic traits were evaluated: total biomass (g), root biomass (g), root length (cm), number of productive tillers, total number of tillers, plant height (cm), average length of spike (cm), average number of grains per spike (g), average weight of grains per spike (g), weight of grains per plant (g), and weight of a thousand grains (g). A complete list of the analyzed agronomic traits with their abbreviations as well as the methods used for their evaluation is provided in **Table 3**

**Table 3:** List of phenotypic traits with description, abbreviations, and measured units.

Trait (unit)	Trait description	Abbrev.
Total biomass (g)	Aboveground and underground biomass determination. Shoots and roots were dried at 60°C for 72 h before weight determination.	**TB**
Root biomass (g)	Underground biomass determination. The root dry mass was obtained using the same drying procedure as that used for the determination of the total biomass.	**RB**
Root depth (cm)	Length of the longest root (distance from the crown to the tip of the root) calculated from 10 randomly selected plants.	**RD**
Number of productive tillers	Number of tillers with fertile spikes.	**NPT**
Total number of tillers	Number of tillers with fertile and nonfertile (without grains) spikes.	**TNT**
Plant height (cm)	Average plant height measured from soil surface to the tip of spike (including awns) at the harvest time. Plant height was measured in a random sample of 10 plants.	**PH**
Spike length (cm)	Length of spike from 10 randomly selected spikes in a plot (without awns).	**LSp**
Number of grains per spike	Number of grains collected from 10 randomly selected spikes in a plot.	**GNSp**
Weight of grains per spike (g)	Average weight of grains per spike, calculated from 10 randomly selected spikes in a plot.	**GWSp**
Weight of grains per plant (g)	Average weight of grains collected from one plant, calculated as average of measurements of grain weight for 10 plants.	**GWp**
Thousand-grain weight (g)	For the measurement of 1000-grain weight, 1000 seeds were taken randomly from each genotype and grain weight were measured.	**TGW**

### Root characterization

At harvest, plants were cut at the soil level and dissected into two main components: roots and shoots. A hose fitted with a fine spray head was used to carefully remove the soil substrate, and the roots (from a 1 m deep piece of soil 1 m deep)were gently teased apart. Because the soil used in this experiment was highly dispersible, excessive manipulation was not required to remove soil particles which can often result in accidental loss of roots.

Electrical capacitance (picofarad—pF) was measured three times at each of the following stages: tillering stage (T1), stem elongation stage (T2), and heading stage (T3). As described by Chloupek et al. (2010) and Středa et al. (2012), capacitances of barley plants were measured using a 380193 Extech LCR meter (Extech Instruments Corporation, USA) at a frequency of 1 kHz. The ground electrode used was a sharpened stainless steel rod (15 cm length and 0.6 cm diameter) inserted up to 10 cm into the substrate, 5 cm away from the stem. The plant electrode was clamped to the stem 1 cm above the substrate level using a 0.5-cm-wide aluminum strip that bent the stem (Wu et al., 2022). A commercial EEG electrode gel was spread under the strip to maintain good electrical contact (Rajkai et al., 2005). Using a standardized methodological protocol, capacitance was measured as described by Středa et al. (2020).

### GBS approach

The GWAS panel was genotyped using the GBS method as described by Poland et al. (2012). For DNA extraction, the barley accessions were grown in a growth chamber at the IPG PAS phytotron facility. Young (2-week-old seedlings) first leaf tissue was obtained from each individual from the plant panel and extraction was carried out using the Wizard Genomic DNA Purification Kit (Promega Corporation, Madison, USA) in accordance with the manufacturer’s protocol. In each tube, 3–5 leaves were pooled per genotype for biological replications. Purity of the DNA was checked using the NanoDrop ND-1000 spectrophotometer (Thermo Fisher Scientific, Waltham, MA), and its integrity was evaluated by agarose gel electrophoresis. Individual DNA samples were diluted to 20 ng/µl with sterile water and then shipped to LGC Genomics, Germany (http://www.lgcgroup.com).

For the GBS analysis, a pilot project was performed in order to assess the optimal enzyme combination, type of library, and number of reads per sample. PstI/MspI were selected as restriction enzymes (ddRAD approach). Briefly, 200 ng of each DNA sample (10 µl volume) was digested with the restriction enzymes and ligated to unique barcode adapters in the main project. Sequencing was carried out on an Illumina NextSeq 500/550 platform (Illumina Inc., USA), and several runs were performed to obtain ~3 M of 75-bp single-ended reads per sample. Data were quality-trimmed, and variant discovery was done with Freebayes v1.0.2-16 software (https://github.com/ekg/freebayes#readme). The obtained variants were further filtered based on the following criteria: (a) the read count for the locus must exceed eight reads, (b) genotypes must have been observed in at least 66% of samples, and (c) the minimum allele frequency across all samples must exceed 5%. No missing SNP data imputation was performed. The IBSC_v2 (https://plants.ensembl.org) genome assembly was used as the reference (Ensembl Plants rel. 49). The sequencing, variant calling as well as SNP filtering were provided by LGC Genomics.

### Statistical analyses

All data were analyzed in Genstat for Windows 21st edition (VSN International, 2019) or R software. Phenotypic data were analyzed in Genstat 21 using analysis of variance (ANOVA) in a mixed linear model (MLM) with fixed effects of year (Y) and random effects of genotype (G) and of G × Y interaction. Heritability was estimated using the method of Cullis et al. (2006). Before analysis outliers were identified by the method of Tukey (1977) in box-and-whisker diagrams and removed from phenotypic data (19 observations).

### LD, population structure, and kinship analysis

The kinship coefficient matrix (based on Dice similarity coefficients) was processed *via* a principal coordinate analysis (PCoA) and used for the hierarchical clustering of accessions to visualize the population structure. LD was estimated for each pair of markers as *r*2 value in linear regression, with one marker used as the response and another one used as the regressor. Based on the LD matrix, the hierarchical clustering of markers was performed in R software using the group average (UPGMA) agglomerative method.

### GWAS analysis

GWAS was carried out by combining the phenotypic data for the 149 barley accessions (genotypic means in individual experiments) and the genotypic data generated using the GBS approach. It was carried out using the method developed by van Eeuwijk et al. (2010) and Malosetti et al. (2013), which allows for the interaction of genetic effects with the environment. The method is based on MLM with the population structure estimated by eigen analysis of the kinship matrix, and with the compound symmetry variance-covariance model used for environmental variation, as implemented in Genstat 21. P values for the effects were corrected for multiple testing using the Benjamini–Hochberg method. An effect was considered significant when the corrected *P* value was lower than 0.05.

### Construction of LD blocks

Based on the significant markers from GWAS, clusters of markers, called LD blocks, were constructed as groups characterized by LD exceeding the mean LD within the chromosome. The second GWAS based on these LD blocks was performed using mean values over all experiments. The analysis of the effects of the LD blocks was carried out using the analysis of covariance in Genstat 21, with the variants within an LD block as the classifying factor and eigen scores of accessions as covariates, with *P* values corrected as in GWAS. Loci for each chromosome were named following the pattern described by N’Diaye et al. (2017) (with minor modification), as a combination of the prefix “LD-b” (LD block), the chromosome number, and the incrementing number index (1 to *N*, *N* being the total number of LD blocks).

### Gene and SNP annotations

Positional and functional interpretation of GBS and GWAS results was carried out in relation to two annotation sources (Gene Ontology and InterPro database) provided in Ensembl Plants and the protein descriptions in the nr protein database in OmicsBox 2.1.2 (BioBam Bioinformatics, 2019). Genes corresponding to SNPs (harbouring them or located nearby) and marker translation effects were found using Ensembl Variant Effect Predictor (VEP) (McLaren et al., 2016).

## Results

### Weather conditions and soil moisture

Monthly precipitation (March–August) ranged from 9.8 to 127.1 mm (**Table 1**). Relatively high rainfall was recorded in two months of 2017 that are crucial for initial plant development (April and May). The highest mean air temperature for all experiments was recorded in the 2018 growing season (April–June). In addition, a decrease in precipitation was recorded in May in the same year (16.6 mm—the lowest mean value for this month from all studied years). The lowest mean precipitation value (2.6 mm) was recorded in April 2020, with rainfall recorded only 6 days a month. In contrast, this growing season was characterized by a relatively high monthly precipitation value in May (47.5 mm). Soil moisture conditions recorded by the FOM/mts instrument in the experimental field are shown in **Table 2**. In 2017, soil moisture was low in the T1 stage compared with that in 2018 and 2020, and then, the mean values increased, reaching the highest of all studied years (13.8%) in the T3 stage. In 2018, the mean soil moisture was high in the initial development phase, but then decreased to 3.1% during the heading stage. The variation in soil moisture during the growing season in 2020 was similar to the pattern observed in 2017, and the highest mean value of soil moisture was observed in the T2 stage during stem elongation. Similarly, the lowest mean soil moisture was observed at the T1 stage in 2020 (7.4%).

### Phenotypic distribution of yield- and root-associated traits

A total of 149 accessions were evaluated in triplicate under field conditions in 2017, 2018 and 2020 at IPG PAS. Eleven agronomic traits were analyzed by GWAS to identify new candidate loci associated with root traits.

**Figure 1:**
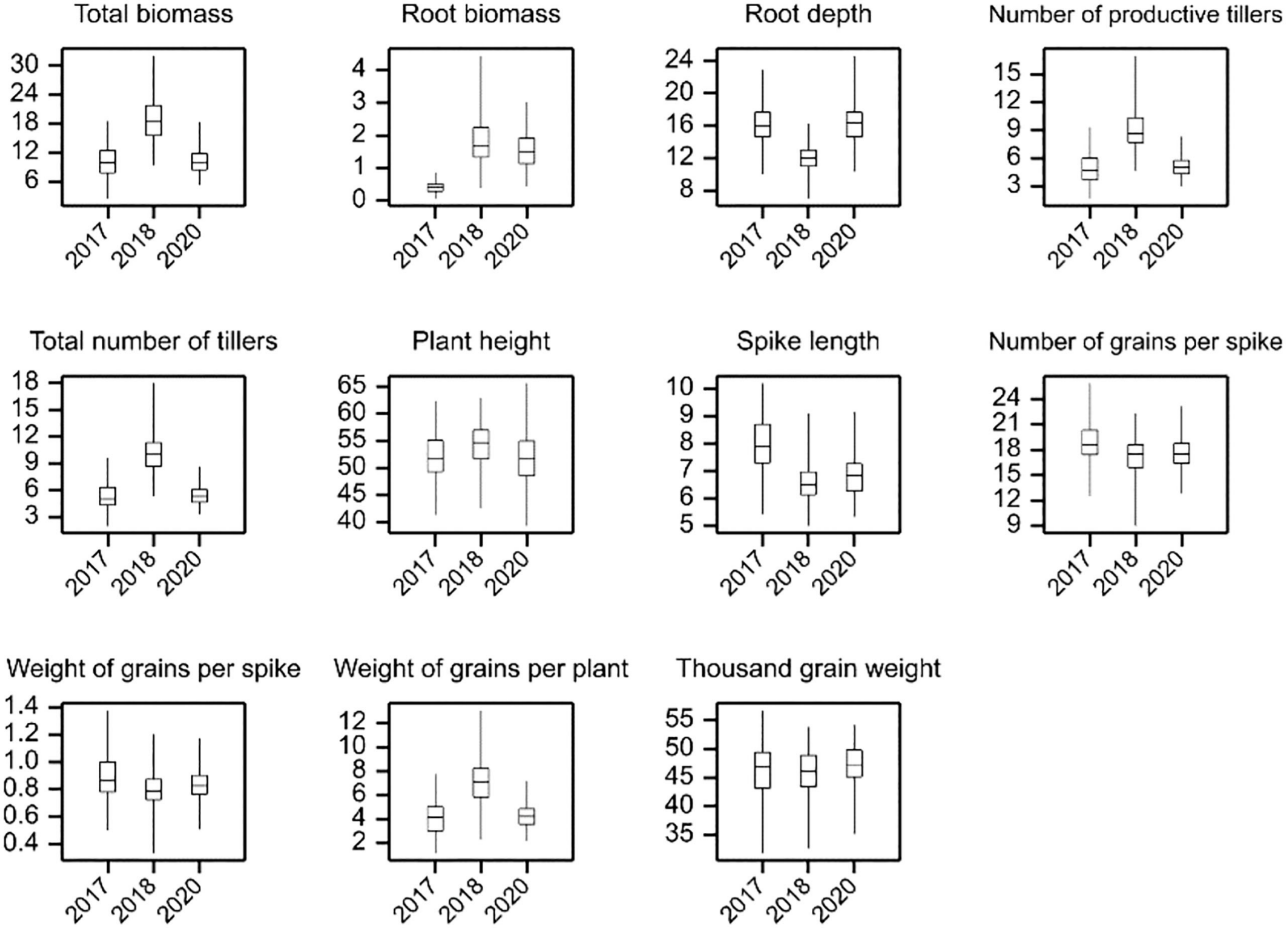
Distributions of genotypic means in experiments.

The distribution of the analyzed traits and the results of the comparison of annual means are shown in **Figure 1** and **Supplementary Table 3**, respectively. The yield-related traits mean values recorded for all genotypes are provided in **Supplementary Table 4**. In the studied years, the highest mean spike length, number of grains per spike, and weight of grains per spike were observed in 2017. On the other hand, in the same year, the mean values of total biomass, root biomass, number of productive tillers, total number of tillers, and weight of grain per plant were lower compared to the next two years of the experiment. A higher mean value of traits related to tillering process (number of productive tillers and total number of tillers) was recorded in 2018. As a result, the total biomass and weight of grains per plant reached the highest mean value in 2018 (18.83 g and 7.16 g, respectively). The mean values of total biomass and root biomass were the highest in 2018, when growing conditions contributed to the better development of the upper part of the plants (shoots). However, the lowest mean root depth was measured in 2018 (11.89 cm), while the mean values for this trait were similar in 2017 and 2020 (16.09 cm and 16.24 cm, respectively). *Electrical capacitance measurements on roots*

**Figure 2:**
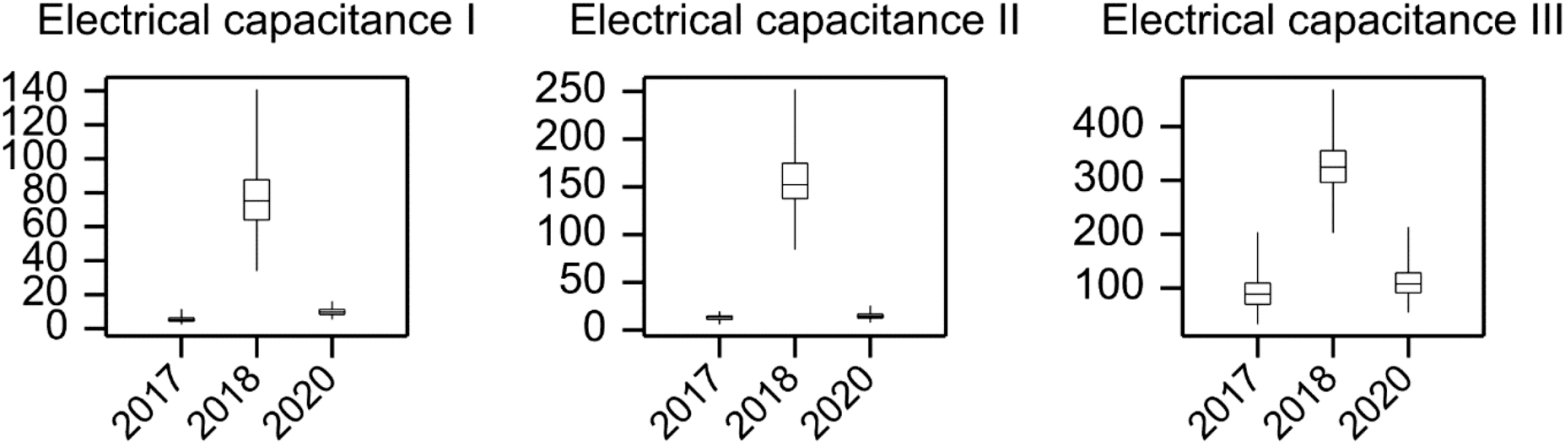
Electrical measurement results for the studied accessions. Electrical capacitance (pico farad – pF) was measured three times - in tillering stage (T1), stem elongation stage (T2) and heading stage (T3).

The results of electrical capacitance measurements that were carried out in all years of the experiments are shown in **Figure 2**. The highest mean values for trait E1-3 were recorded in 2018. Slightly lower E1-3 mean values were recorded in 2017, when root biomass was lowest, compared to the mean values recorded in 2020. Due to large differences between years in terms of both mean values and dispersion of genotypic means, electrical capacitance observations were centered and normalized within the years using observed means and standard deviations.

### Analysis of variance and correlations between root- and yield-related traits

ANOVA based on data from three years of evaluation showed that the variation among genotypes was relatively larger for the traits PH and TGW than for the traits RB and GWSp (**Supplementary Table 5**). The estimates of the genotypic variance were smaller than the corresponding G × Y interaction variance for almost all traits (except for PH). Root traits (RB and RD) were most affected by G × Y variance. Broad-sense heritability estimates ranged from 7.0% (for RB) to 60.6% (for PH). Strong positive correlations (*P* value < 0.05) were observed between traits associated with tillering (NPT and TNT) and TB (correlation = 0.93 and 0.91, respectively) (**Table 4**). TB also showed a significant correlation with GWp (0.96). A negative correlation was found between RD and traits related to plant biomass (TB and RB) and between RD and tillering traits (NPT and TNT). A weak positive correlation was recorded between TB and both E1 and E2 (0.27 and 0.29, respectively).

**Table 4:** Correlations between the studied traits of GWAS panel accessions (*P* value < 0.05).

Trait name (abbrev.)	TB	RB	RD	NPT	TNT	PH	LSp	GNSp	GWSp	GWp	TGW	E1	E2	E3
TB	1													
RB	0.62	1												
RD	−0.42	−0.22	1											
NPT	0.93	0.57	−0.45	1										
TNT	0.91	0.54	−0.49	0.97	1									
PH	0.36	0.26		0.21	0.21	1								
LSp	−0.24	−0.42	0.28	−0.42	−0.41	0.2	1							
GNSp		−0.14	0.2	−0.21	−0.22	0.25	0.67	1						
GWSp			0.23	−0.22	−0.26	0.22	0.6	0.87	1					
GWp	0.96	0.57	−0.37	0.91	0.87	0.31	−0.22	0.12	0.15	1				
TGW			0.17	−0.12	−0.17		0.19	0.24	0.68	0.14	1			
E1	0.27	0.14		0.26	0.24	0.13				0.3		1		
E2	0.29	0.14		0.29	0.25	0.18				0.32		0.59	1	
E3						0.16						0.21	0.3	1

### Genotyping and population structure

**Figure 3:**
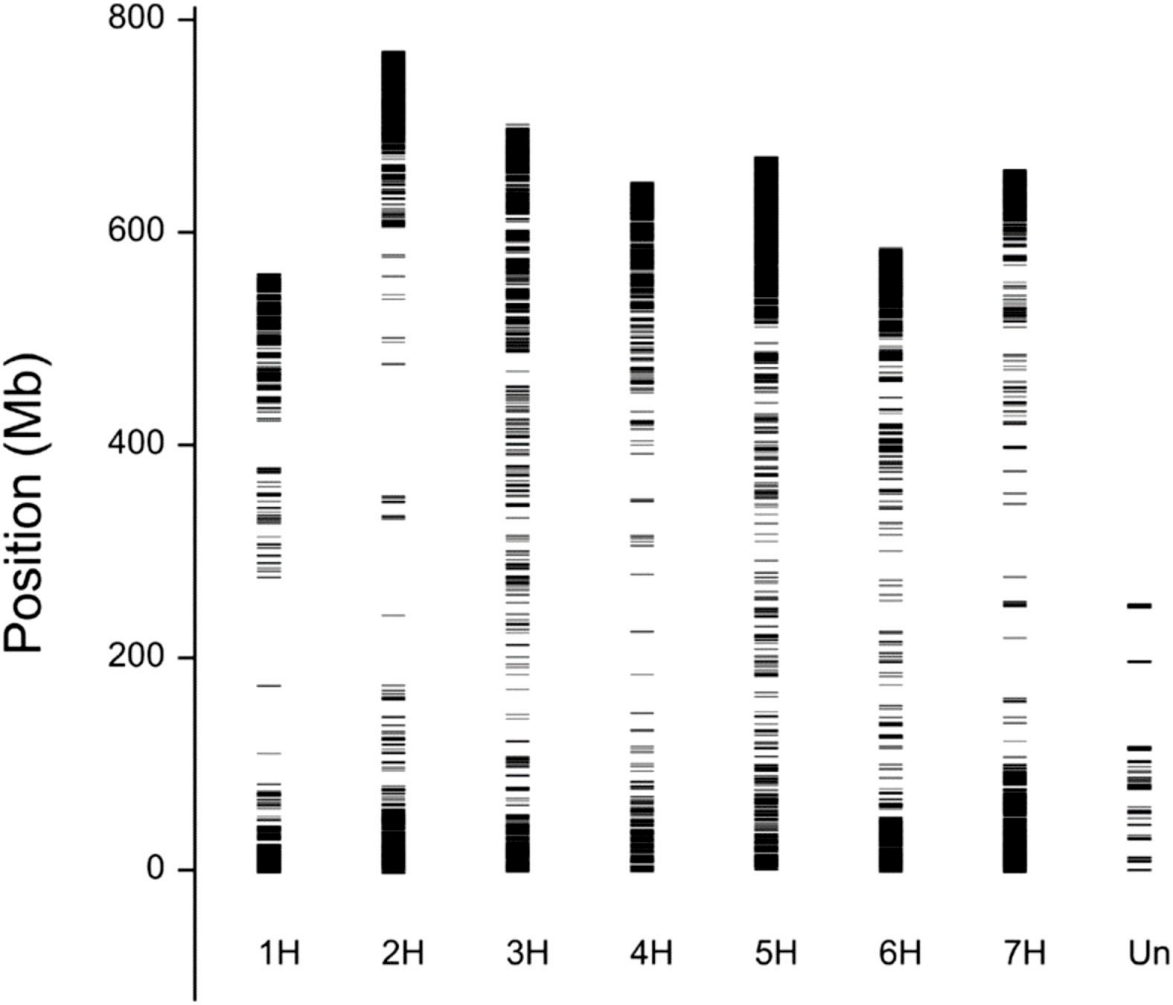
Physical mapping of 5739 SNP markers on barley chromosomes. Marker coverage is lower near the centromere and better further from the centromere, closer to telomere. The reason is not in the method, but in the fact that polymorphisms occur more often closer to the ends of the chromosomes than near to the centromere.

**Figure 4:**
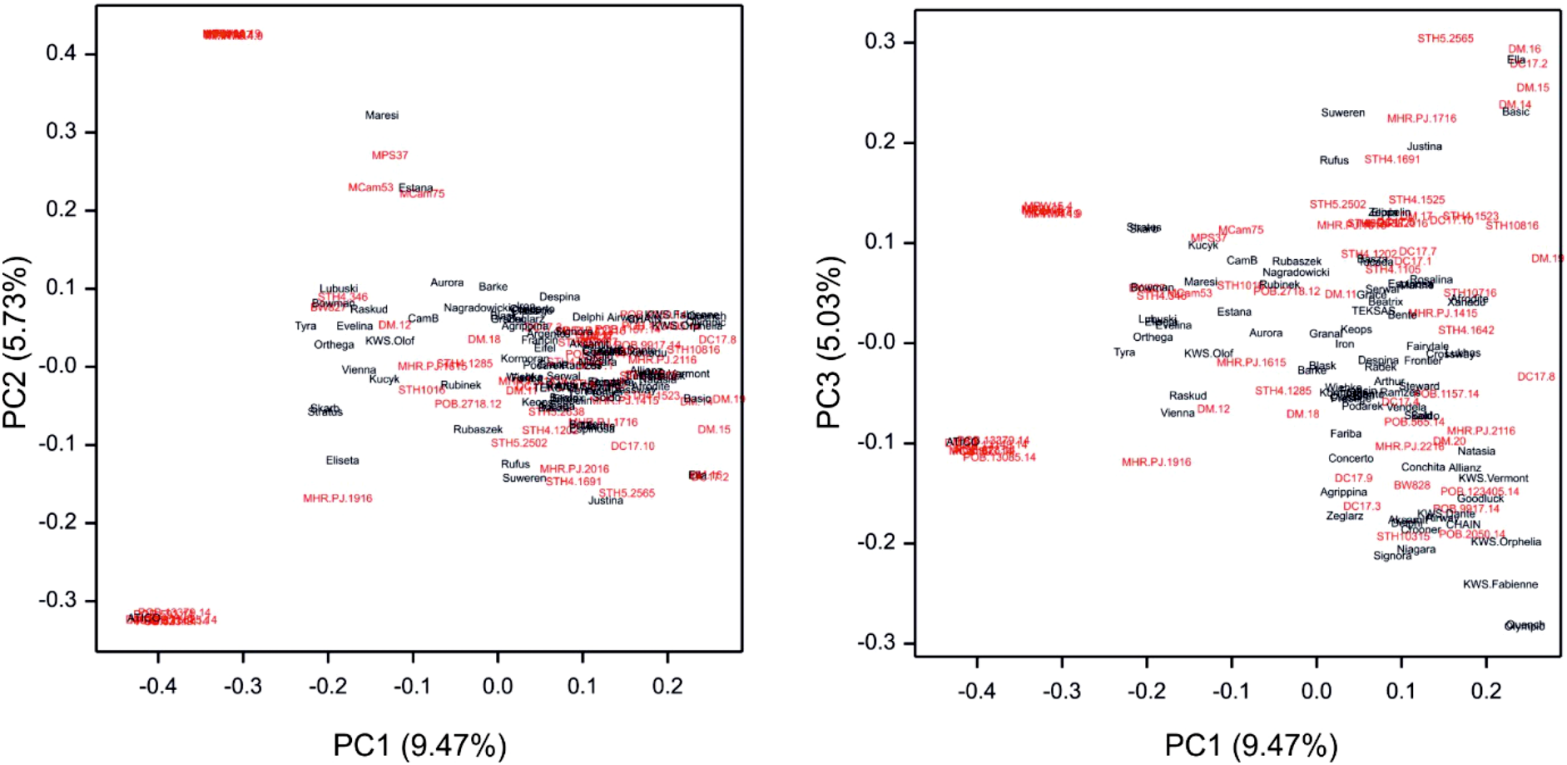
Visualization of the population structure by PCoA of the kinship coefficient matrix for accessions (cultivars – black, breeding lines – red) based on SNP markers. Cultivar accessions were grouped on the right side and evenly distributed groups of breeding lines overlapped with the subgroups of cultivars.

Genotyping of the GWAS panel using the GBS method revealed a total of 5739 SNPs (**Supplementary Table 6**). The physical location (**Figure 3**) of the SNP markers shown their uneven distribution on and within the barley chromosomes. Wider gaps in marker coverage were observed on chromosomes 1H, 2H, 4H, and 7H, whereas the distribution of SNP markers was more uniform on chromosomes 3H, 5H, and 6H. The number of SNP markers on individual chromosomes ranged from 569 (chromosome 1H) to 977 (chromosome 7H). The population structure (**Figure 4**) was visualized by PCoA on the kinship (coancestry coefficients) matrix of accessions that were derived from all 5739 SNP markers. PCoA did not divide the accessions into clear clusters; however, some subgroups were observed. Cultivar accessions were mainly grouped on the right side (positive values of PC1), and evenly distributed groups of breeding lines overlapped with the subgroups of cultivars. PCoA separated some breeding lines (offspring with parents) from the rest of the genotypes. As shown in **Supplementary Figure 1**, the dendrogram obtained by hierarchical clustering based on the kinship matrix confirmed the results of PCoA: the barley breeding lines and cultivars that were used as parents/donors at some stage of the breeding process formed groups (e.g., CamB and the progenies such as MCam53, MCam75, and MPW15.4).

### Genome-wide association analysis

**Figure 5:**
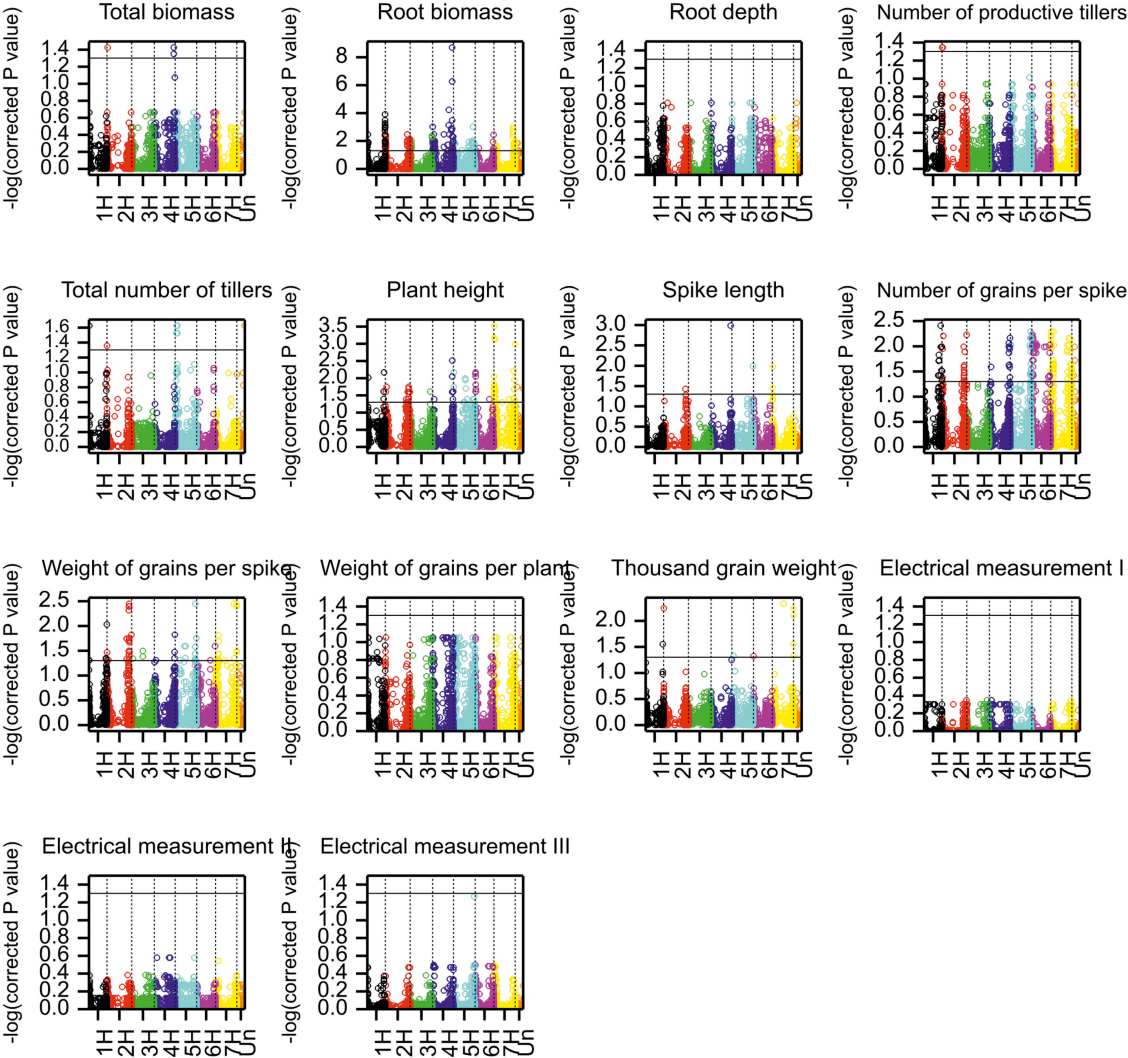
Manhattan plots for the associations of markers with phenotypic traits. Black horizontal line indicates −log(0.05), the significance threshold for log-transformed Benjamini-Hochberg corrected *P* values.

A total of 690 marker–trait associations (MTAs) were detected for 8 of 14 analyzed traits at a significance threshold (corrected *P* value) of 0.05 (**Table 5**). Manhattan plots depicting the significant SNP markers above the threshold are shown in **Figure 5**. The highest number of associations was identified for RB a total of 246 MTAs distributed across the whole barley genome, with the majority (61) on chromosome 4H and the lowest on chromosome 6H (**Supplementary Table 7**). Of the 690 MTAs, 91 were linked to more than one trait (**Supplementary Table 7**). Five SNP markers on chromosome 7H were associated with four agronomic traits (**Table 6**): RB, GNSp, GWSp, and TGW (all with negative effects). The marker SNP 2H_759960282 located on chromosome 2H had a positive effect on RB, PH and GNSp, and a negative effect on GWSp. The lowest percentage of significant markers demonstrating interaction with the environment was for TGW (29%), and the highest for TB and LSp (100%), however it was for few associations (3 and 6, respectively). It is worth to mention that 94% of significant marker effects for RB (from 246 associations) were showing interaction with the environment (**Table 5**), which is in agreement with low heritability and large interaction for this trait revealed by ANOVA; for majority of markers this interaction could be attributed to a larger allelic effect in 2018 (**Supplementary Table 7**).

**Table 5:** Characteristics of allelic effects for SNP markers significantly associated with phenotypic traits.

Trait	No. of associations	Significant effects		Negative significant effects		Positive significant effects		Percent of significant associations with interaction with environment
		Negative	Positive	Min	Max	Min	Max	
Total biomass	3	1	2	−0.2875	−0.2875	0.7506	0.9266	100%
Root biomass	246	116	130	−0.0951	−0.0001	0.0001	0.0791	94%
Number of productive tillers	2	1	1	−0.2579	−0.2579	0.8607	0.8607	50%
Total number of tillers	11	0	11			0.6653	1.2714	91%
Plant height	88	46	42	−3.1966	−0.0668	0.001	3.5334	73%
Spike length	6	2	4	−0.5873	−0.3122	0.3456	0.5079	100%
Number of grains per spike	222	118	104	−1.5758	−0.0071	0.0062	1.3028	91%
Weight of grains per spike	98	51	47	−0.1547	−0.0032	0.0031	0.1717	68%
Thousand grain weight	14	10	4	−3.0049	−1.87	0.5245	3.1386	29%

**Table 6:** Markers significantly associated with four phenotypic traits and their allele substitution effects.

Marker	RB	PH	GNSp	GWSp	TGW
2H_759960282	0.027	0.001	0.196	−0.036	
7H_655557437	−0.054		−0.401	−0.065	−2.655
7H_655662129	−0.060		−0.282	−0.058	−2.426
7H_656716470	−0.056		−0.377	−0.063	−2.474
7H_656716493	−0.056		−0.377	−0.063	−2.474
7H_656717073	−0.050		−0.325	−0.063	−2.636

### Distribution of LD blocks

Genetic dissection of associations for complex traits was performed using an LD-based haplotype approach. As shown in **Supplementary Table 8**, a total of 59 LD blocks containing 2–32 SNPs were created as described in the Materials and Methods section. The allele combinations were further filtered based on the following criteria: the minimum number of occurrences of a given combination is eight and LD blocks with at least two combinations remain for further analysis. Therefore, the number of allele combinations varied from two to six for the LD blocks. The highest number of LD blocks was identified on chromosome 2H, whereas the lowest number of blocks on chromosome 3H excluding Un (**Supplementary Table 9**). The largest number of LD blocks was associated with trait PH (12), whereas the lowest number was linked to TGW (2). Seven LD blocks were associated with each of the traits RB, GNSp, and GWSp. No associated LD blocks were identified for the traits TB, RD, NPT, TNT, LSp and GWp.

### Yield-related traits

#### Plant height

Twelve LD blocks showed significant association with PH. Their distribution on chromosomes was as follows: one on 1H, three on 2H, two on 4H, one on 6H, four on 7H, and one on Un. For the LD-b_7H_55 block, one SNP combination (T/T C/C C/C A/A A/A C/C) resulted in the largest increase in PH (3.09 cm) among three associated marker combinations. This combination was observed in the smallest number of accessions – 9 out of 135. The largest decrease in PH (4.01 cm) was identified for the combination G/G G/G C/C C/C A/A G/G C/C G/G C/C A/A of LD-b_4H_31 block.

#### Grains number per spike

Seven LD blocks were associated with GNSp, three of which were located on chromosome 2H, three on chromosome 4H, and one on chromosome 7H (LD-b_7H_58). Two LD-b_7H_58 SNP combinations resulted in the largest decrease in GNSp (1.29–1.31 g). LD-b_4H_31 included six SNP combinations, of which the combination C/C G/G C/C C/C A/A G/G C/C G/G C/C A/A resulted in a GNSp increase of 0.70 g.

#### Grains weight per spike

Six associations (except one on Un) were identified for GWSp on chromosomes 2H, 3H, and 7H. The SNP combination of the block LD-b_7H_58, which included nine accessions, showed the largest negative allelic effect (−0.11 g). The marker combination (T/T G/G) linked to the GWSp block (LD-b_2H_17) resulted in the largest increase of GWSp (0.1 g).

#### Thousand-grain weight

Two LD blocks were detected for this trait. The largest negative allelic effect (−3.08 g) was found for one of two SNP combinations of LD-b_1H_3. The SNP combination (A/A A/A) of LD-b_2H_6 resulted in the largest increase of TGW (1.56 g).

### Root-related traits

#### Root biomass

Seven LD blocks significantly associated with RB were identified on chromosomes 2H, 4H, 5H, and 7H. In general, eight SNP combinations with positive allelic effects on RB were detected. Combination G/G A/A C/C of LD-b_4H_26 showed the largest allelic effect, resulting in 0.41g increase in RB compared to the general mean RB value of all accessions. This combination was identified in 8 of 146 accessions identified for this block. Twelve combinations with negative effects on the studied trait were identified with the largest decrease in RB (−0.25 g) for the SNP combination T/T G/G A/A of LD-b_7H_58 (**Supplementary Table 9**).

#### Marker translation effects on root biomass

VEP analysis revealed three SNP markers with both significant effects on RB and high translation effects (**Supplementary Table 7**). 1H_551858749 and 2H_21581482 markers showed significant positive effects on RB. The last SNP with a high marker translation (negative) effect was located in chromosome 6H (6H_551431916).

#### Root-biomass-associated LD blocks collocating with additional traits

**Figure 6:**
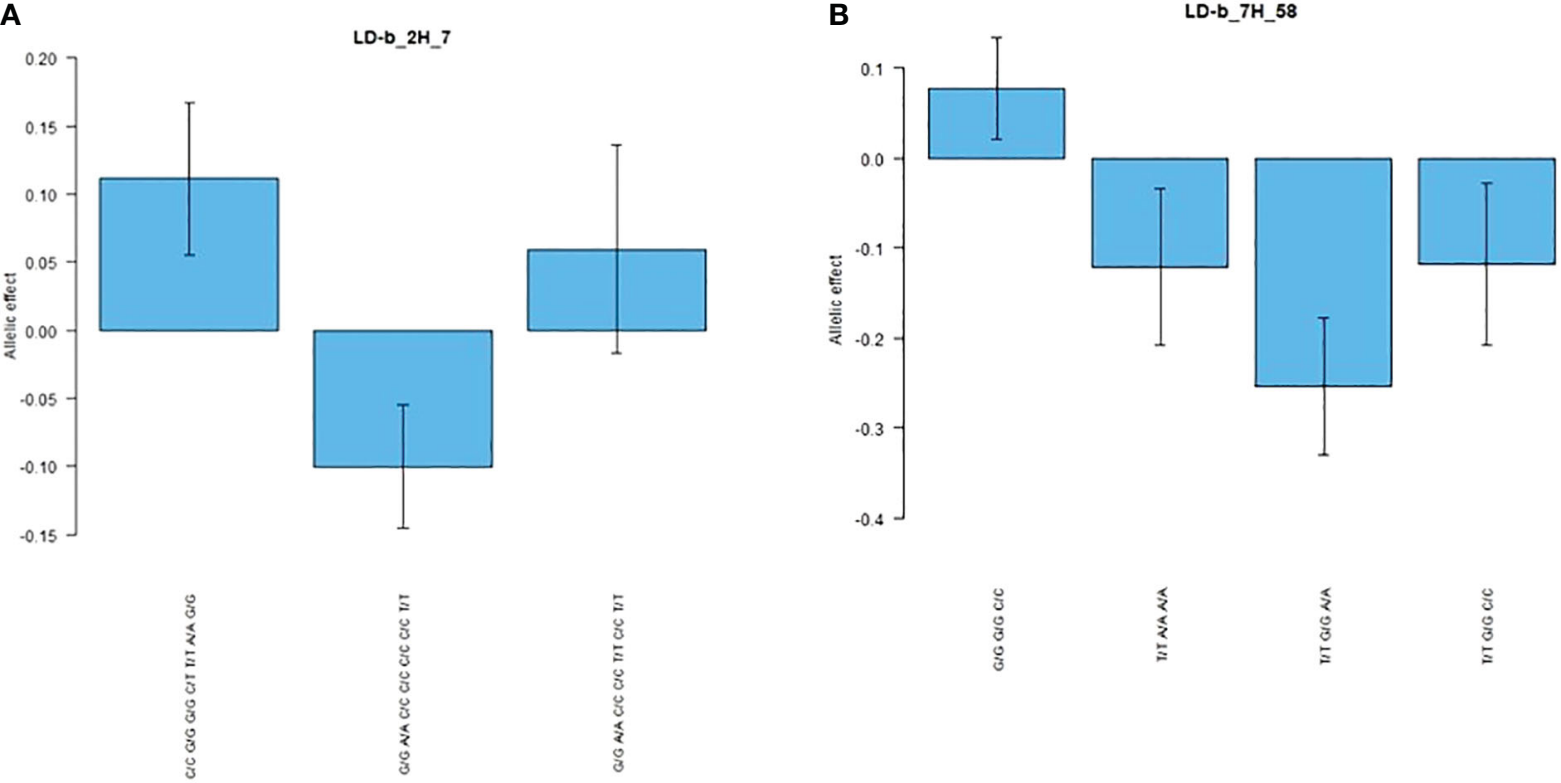
Allelic effects of genotypic combinations within **(A)** block LD-b_2H_7 and **(B)** block LD-b_7H_58 for RB trait.

As shown in **Table 7**, two RB markers that were significantly associated with LD blocks were collocated with additional traits: LD-b_2H_7 was collocated with PH, while LD-b_7H_58 with PH, GNSp and GWSp. LD-b_2H_7 associated with RB also collocated with PH, whereas on chromosome 7H, an SNP combination (G/G G/G C/C) of block LD-b_7H_58 was observed, resulting in an increase in all four traits (RB, PH, GWSp and GNSp). The allelic effects of SNP combinations within LD-b_2H_7 and LD-b_7H_58 are shown in **Figure 6**. The allelic effects of SNP combinations identified for LD blocks related to both RB- and yield-related traits were larger than those defined for individual SNP markers. Moreover, in some cases (e.g., LD-b_7H_58), the SNP combination had the opposite allelic effect compared with the effects of the individual markers. For the vast majority of these SNP markers, positive interaction with the environment was recorded (e.g., LD-b_2H_7—interactions were found for six of seven markers).

**Table 7:** Characterization of significant root-related LD blocks also associated with other traits.

LD block name	SNP combinations	No. of accessions	Allelic genotypic effects			
			RB	PH	GNSp	GWSp
LD-b_2H_7	G/G A/A C/C C/C C/C C/C T/T	54	−0.1	−1.26	−	−
LD-b_2H_7	C/C G/G G/G C/T T/T A/A G/G	38	0.11	0.83	−	−
LD-b_2H_7	G/G A/A C/C C/C T/T C/C T/T	20	0.06	1.83	−	−
LD-b_7H_58	T/T A/A A/A	10	−0.12	−0.09	−0.15	−0.02
LD-b_7H_58	T/T G/G A/A	15	−0.25	−3.15	−1.29	−0.06
LD-b_7H_58	T/T G/G C/C	9	−0.12	−2.44	−1.31	−0.11
LD-b_7H_58	G/G G/G C/C	78	0.08	0.9	0.42	0.03

#### Gene annotations of SNPs associated with RB

The results obtained from functional annotation using different annotation tools for all GWAS results are presented in **Supplementary Table 7**. Data from three annotation sources, namely Gene Ontology, nr database and InterPro database, provided in Ensembl Plants were collected for SNPs with significant associations for RB with LD blocks (collocated with other yield-related traits; **Table 8**) and for SNPs with both significant positive effects for RB and high translation effects (**Table 9**). The annotations belonged to several functional categories, namely protein kinase, transcription factors, or other stress-related candidates, while some of them had unknown functions. For some SNP markers, identical annotations were recorded due to their linkage to the same gene ID.

**Table 8:** Selected gene annotations for LD blocks linked concurrently to root- and yield-related traits.

LD block	Markers in block	Gene ID	InterPro	Gene Ontology	nr database
LD-b_2H_7	2H_14745634	HORVU2Hr1G007220	–	–	–
	2H_15019351	HORVU2Hr1G007340||HORVU2Hr1G007350	IPR000719|Protein kinase domain; IPR001245|Serine-threonine/tyrosine-protein kinase, catalytic domain; IPR008271|Serine/threonine-protein kinase, active site; IPR011009|Protein kinase-like domain superfamily; IPR017441|Protein kinase, ATP binding site||IPR039274|Flowering-promoting factor 1	C:integral component of membrane;C:membrane;C:plasma membrane;F:ATP binding;F:nucleotide binding;F:protein kinase activity;F:protein serine/threonine kinase activity;P:cell surface receptor signaling pathway;P:protein phosphorylation||P:regulation of flower development	Wall-associated kinase-like 1||predicted protein
	2H_15019363				
	2H_15036816				
	2H_16421680	–	–	–	–
	2H_16946037	HORVU2Hr1G008240	IPR000719|Protein kinase domain;IPR000858|S-locus glycoprotein domain;IPR001480|Bulb-type lectin domain;IPR011009|Protein kinase-like domain superfamily;IPR017441|Protein kinase, ATP binding site;IPR036426|Bulb-type lectin domain superfamily	F:ATP binding;F:protein kinase activity;P:protein phosphorylation;P:recognition of pollen	S-domain receptor-like protein kinase Serine/threonine-protein kinase
	2H_16946042				
LD-b_7H_58	7H_600891887	HORVU7Hr1G099520	IPR000719|Protein kinase domain;IPR001245|Serine-threonine/tyrosine-protein kinase, catalytic domain;IPR001611|Leucine-rich repeat;IPR003591|Leucine-rich repeat, typical subtype;IPR008271|Serine/threonine-protein kinase, active site;IPR011009|Protein kinase-like domain superfamily;IPR013210|Leucine-rich repeat-containing N-terminal, plant-type;IPR017441|Protein kinase, ATP binding site;IPR032675|Leucine-rich repeat domain superfamily;|	C:integral component of membrane;C:membrane;F:ATP binding;F:kinase activity;F:nucleotide binding;F:protein binding;F:protein kinase activity;F:protein serine/threonine kinase activity;F:transferase activity;P:phosphorylation;P:protein phosphorylation	C:integral component of membrane;C:membrane;F:ATP binding;F:kinase activity;F:nucleotide binding;F:protein binding;F:protein kinase activity;F:protein serine/threonine kinase activity;F:transferase activity;P:phosphorylation;P:protein phosphorylation
	7H_604533359	ENSRNA049488629||ENSRNA049488633	U2 spliceosomal RNA||U2 spliceosomal RNA (gene description)	–	–
	7H_607372612	HORVU7Hr1G101010	IPR006566|FBD domain;IPR032675|Leucine-rich repeat domain superfamily;IPR036047|F-box-like domain superfamily	F:protein binding	F:protein binding

**Table 9:** Selected gene annotations for SNPs with both significant positive effects for RB and high translation effects.

LD block	Markers with high translation effect in block	Gene ID	InterPro	Gene Ontology	nr database
LD-b_1H_4	1H_551859749	HORVU1Hr1G092980	IPR013057|Amino acid transporter, transmembrane domain;IPR021480|Probable zinc-ribbon domain, plant;|	C:integral component of membrane;C:membrane	Putative GABA transporter 2
LD-b_2H_10	2H_21581482	HORVU2Hr1G010990	IPR000425|Major intrinsic protein;IPR022357|Major intrinsic protein, conserved site;IPR023271|Aquaporin-like;IPR034294|Aquaporin transporter	C:integral component of membrane;C:membrane;C:plasma membrane;C:plasmodesma;C:vacuole;F:channel activity;F:water channel activity;P:transmembrane transport;P:water transport	Plasma membrane intrinsic protein

## Discussion

In our previous studies conducted under field conditions, we used linkage mapping to map QTL to determine quantitative traits in spring barley (Ogrodowicz et al., 2020). It is, however, well known that fine-mapping QTL using a linkage analysis needs a population of thousands of individuals (He et al., 2017) and that the limited polymorphic loci between the two parents influence mapping accuracy. Although our accession panel included a sufficient number of plants, we additionally increased the efficiency of single-marker GWAS by using an LD-based haplotype approach to examine root- and yield-related traits in our spring barley panel. In the literature, there are many papers on GWAS for less than 150 plant genotypes, e.g., Ain et al. (2015) used 123 Pakistani historical wheat cultivars, Valluru et al. (2017) used 130 diverse wheat elite lines and landraces, Arora et al. (2017) used 114 wild wheat accessions, Lehnert et al. (2018) used 94 bread wheat genotypes, Ma et al. (2018) used a panel of 66 elite wheat genotypes, and Rohilla et al. (2020) used 94 deep-water rice genotypes. In recent years, association mapping has been widely used to detect quantitative trait loci (Reinert et al., 2016; Abdel-Ghani et al., 2019). In the present study, we used a differentiated accession panel comprising cultivars, recombinant inbred lines, breeding lines, and backcross lines. Nevertheless, the population structure analysis revealed two subgroups, which were clustered mainly in terms of origin; each group contained the donors (cultivars or mutants) used in the breeding processes - e.g., CamB and Maresi – parents in the breeding process of two RILs, namely Mcam53 and Mcam75; Bowman and the backcross line – BW827 (Mikołajczak et al., 2022).

The present study aimed to investigate the role of root architecture in plants grown under natural field conditions. We used the LD-based GWAS to investigate root- and yield-related traits in the spring barley panel. Although several studies have used GWAS to explore the diversity of barley (Nandha and Singh, 2014; Tsai et al., 2020), genetic analysis of root traits has not been commonly performed because of the difficulty in their phenotypic evaluation. Most research on this subject is conducted under greenhouse or growth chamber conditions because of the constraints in conducting field studies (Paez-Garcia et al., 2015). To the best of our knowledge, our study is the first to investigate the major variations in root and related shoot traits by using LD block-based GWAS in spring barley accessions grown under field conditions. Considering the difficulties in root phenotyping in the field, we also used the nondestructive root evaluation method to understand the root structure of the studied accessions.

Because of their ability to retain water and nutrients, roots and their architecture are considered the most important parameters for plant productivity and adaptation to drought stress. The process of development of roots of the same cultivar varies under different environmental conditions such as soil type, water and nutrient availability, and crop management practices (Motzo et al., 1992; Nevo and Chen, 2010; Wasson et al., 2012). Under low water conditions or periods, root plasticity can highly influence crop performance. Some cultivars can better respond to drought through additional root growth than other cultivars (Ehdaie et al., 2012). In some cases, the total root biomass of plants in a water-scarce environment might even increase as compared to that under well-watered conditions (Sharp and Davies, 1985; Thornley, 1998). Although several studies have been conducted to analyze the role of root architecture in different plant species (Ruangsiri et al., 2021; Wu et al., 2022), there remains a gap in our knowledge, particularly regarding how roots are affected by environmental factors (Freschet et al., 2013).

### Phenotypic evaluation

In this study, phenotypic evaluation showed significant variations in root- and yield-related traits across the three studied years, thus indicating broad genetic and phenotypic variance within the studied GWAS panel. In 2017, under favorable rainfall conditions, plants did not develop many tillers; however, weather conditions contributed to the development of longer spikes, because of which high mean values of GWSp were recorded. In 2018, the mean values of traits that contribute to yield (TB, RB, NPT, GWp, etc.) and tillering showed a significant increase. In the same year, plants developed much higher root biomass than that in the other two years. Concurrently, the lowest mean root depth values were observed in 2018, which indicated that the studied plants did not develop long roots to search for water resources in the soil. The soil moisture content recorded at the tillering stage (T1) was the highest, which directly contributed to the development of tillers. Under these conditions, plants did not develop deep root systems as the existing root structure allowed sufficient access to water resources. In 2018, a rapid decrease in soil moisture content was recorded only in the heading phase, when the studied plants were mainly in the maturation stage. According to the “balanced growth” hypothesis (Bloom et al., 1985), some plants respond to drought by stimulating or maintaining root growth while reducing shoot growth. In other words, plasticity may be a critical strategy adopted by plants to respond to unstable water conditions. Deep roots that facilitate water acquisition from deeper areas of soil may be particularly important for smaller plants such as wheat, rice, and barley; however, this aspect is generally beneficial for plants that grow under limited soil water conditions in agricultural and natural systems (Henry et al., 2011). Even if the soil becomes completely dry at the surface, water may be available deeper in the profile, which is inaccessible to many agricultural species. Consequently, these species must develop deeper root systems to access this water profile. In contrast, high soil water content or soil density may reduce the root elongation rate and the number of lateral roots, which may also be attributed to a reduction in shoot growth (Bengough et al., 2011), as indicated by the results of the present study in relation to the plant development model observed in 2017.

Content and weather conditions provided information on the dynamic changes in water availability during the course of the field study. The average monthly precipitation values varied across the three years of our investigation; for example, extremely low number of days with rainfall were recorded in 2020. These differences in monthly weather conditions across the year influenced the yield performance of the studied plants to some extent. The studied accessions were not as stable as those grown under greenhouse conditions, but they were rather subject to dynamic, rapid changes in the environment. The monitoring of soil moisture content showed that spring drought occurred under field conditions in both 2017 and 2020, even though rainfall was relatively high in 2017. However, as several constraints limit water use, the use of water resources by plants is not solely dependent on rainfall (e.g., Gavrilescu, 2021).

In this study, the highest values for electrical measurements were observed in 2018, when accessions developed many tillers and both total and root biomass increased. As previously reported, a strong correlation exists between the electrical capacitance of the root and the weight of the root system in crops (Cseresnyés et al., 2018). Therefore, the probability of selecting a larger root system based on electrical capacitance is high. In recent years, root system size (measured by electrical capacitance) has been considered as a criterion for selecting drought-tolerant genotypes in crops, such as spring barley (Chloupek et al., 2010; Svačina et al., 2014) and winter wheat (Abdel-Ghani et al., 2013). We also applied the electrical capacitance method to evaluate the root architecture of barley grown under field conditions. Brown et al. (2012) showed that roots with a diameter of 0.25 mm accounted for almost 95% of the total root length. Thus, a rapid, efficient, and nondestructive method must be used for root system assessments in order to investigate the relationship between root properties and yield. An undeniable advantage of the electrical capacitance method is that it can measure even the finest root structures (root hairs) because it is based on biophysical principles.

Almost all common methods used to observe the below ground parts of the plant have their own advantages and disadvantages (Freschet et al., 2021). Some approaches employed wet-sieving method in a greenhouse (Hudek et al., 2022) and under field conditions (Gan et al., 2009) or monolith excavation method under field condition (Mamedov and Husiyev, 2022). In this study, we excavated the monolith of soil (1 m deep and 0.5 m width) containing a representative root zone of each genotype, since, as reported for barley, wheat or corn, the most of plant roots are concentrated in depth level ∼50-100 cm (ARD, 2013). Roots were then carefully extracted from the soil by washing and drying following the procedure of Gan et al. (2009) (with minor modifications) procedure and then measurements were performed. The results from ANOVA in our study showed that root-associated traits were significantly affected by the G × Y interaction, which was confirmed by the relatively low broad-sense heritability values estimated for RB and RD. Some previous studies have shown that many root-related traits such as root biomass and root length remain relatively stable across different environments (Pantalone et al., 1996; Liu et al., 2005). On the other hand, the root system of plants is influenced by management and environmental conditions and is dependent on the genotype, as plants respond to nutrient limitation and water deficit in soil by increasing their root biomass, and thus the ratio of root to shoot biomass (Abdel-Ghani et al., 2013). Moreover, previous studies have indicated that traits associated with root traits, such as RB and RD, regulate the root architecture, which is essential for successful soil exploration (Falik et al., 2012). In our study, root development was influenced by both dynamic changes in weather and soil moisture conditions and genetic interactions.

The results of the study showed a negative correlation between RD and traits linked to plant biomass (TB and RB), which is consistent with the observations of Sierra Cornejo et al. (2020) who reported that plants develop longer roots to explore the deepest layers of soil where water can still be available. Our study also found a strong positive correlation between RB and traits linked to tillering. Excessive tillering resulted in the formation of a higher number of nodal roots, suggesting that the dependence of the development of shoot and root is in line with the strong positive relationship recorded for RB and NTP with TNT. Among the studied traits, a subgroup of plants can be identified with a similar RB—correlation rate with other traits. This phenomenon confirms the role of genetic background in the development of root architecture.

### Marker–trait associations

In this study, the results of a LD-based GWAS for 11 phenotypic traits related to plant morphology, grain quality, and root apparatus in a collection of spring barley were presented. Small association panels were observed to increase both type 1 and type 2 error rates, resulting in the failure to detect true associations while producing a higher rate of false-positive associations (Sesia et al., 2021). The number of genotypes used in this study was similar to the one used for other genomic studies, yet we used a conservative approach of GWAS to reduce false associations. We used an MLM that corrects for population structure using kinship and detected 690 MTAs. The population structure was consistent with the known differences in pedigree and the source of the breeding program. In this study, a large proportion of MTAs (246) were found for the trait RB. These associations were distributed across all chromosomes; this confirms the quantitative architecture of the studied traits. Recently, the importance of root weight has been demonstrated in various studies (e.g., Mandozai et al., 2021; Zhao et al., 2021). Several investigations have shown that a deeper root system is influenced by dry weight and total length of the roots, which is important for improving drought tolerance as well as the final yield under drought stress in crops such as soybean (Hudak and Patterson, 1995) and rice (Suji et al., 2012). Root biomass accumulation is beneficial for drought escape as it could allow efficient uptake of soil water and nutrients (Danakumara et al., 2021). In general, plants growing in nutrient-deficient soils have higher root-to-shoot ratios, suggesting that more resources are allocated to root development, which enables the extraction of nutrients from a deeper and wider zone (Shen et al., 2018). Twenty-nine MTAs observed for RB were simultaneously associated with other yield-related traits. The association between markers and multiple root traits has also been demonstrated in other studies (Long et al., 2013; Chen et al., 2016). These results are well established as a result of the polygenic nature of the investigated traits and confirm the vital role of pleiotropy in synergistic biomass accumulation of roots in barley plants. The finding that MTAs detected in the studied barley panel are simultaneously responsible for root biomass and some yield-related traits suggests that root traits are controlled by multiple loci and highlights the importance of these genomic regions in root growth and architecture.

### Analysis of LD blocks

LD blocks were constructed based on the average LD extent in our material for each chromosome. The LD-based approach has been shown to be a reliable tool for analyzing quantitative traits in different crops (N’Diaye et al., 2018). An advantage of this method is that it avoids an arbitrary or suggestive number of markers included in the block. Lorenz et al. (2010) and Contreras-Soto et al. (2017) have shown that the use of haplotype information allows identifying marker–phenotype associations and facilitates the genetic analysis of loci underlying complex traits. In our study, out of the significantly associated LD blocks, 58 contained two or more SNPs that are previously detected in GWAS. This indicates the advantage of haplotypes in the detection of multiple DNA variants. Seven LD blocks associated with the RB trait were found on chromosomes 2H, 4H, 5H and 7H. The largest number of RB blocks was located on chromosome 2H (three LD blocks). This is consistent with the study of Jia et al. (2019) which demonstrated the role of the region on chromosome 2H in shaping root architecture. In addition, these authors identified QTL on chromosomes 2H, 4H, 5H and 7H that were simultaneously associated with root- and yield-related traits. The identification of RB-associated LD blocks on the same chromosome in accordance with previous studies could confirm the associations found in the present study. QTLs that determine root fresh weight were previously found on chromosomes 1H, 2H and 5H (Xu et al., 2012; Broughton et al., 2015). Regions on chromosome 7H have been primarily identified as associated with root length (Ain et al., 2015; Xue et al., 2017), however, associations between the regions on chromosome 7H linked to root biomass have also been reported (Arifuzzaman et al., 2014; Arifuzzaman et al., 2017).

In the present study, two root-related LD blocks were of particular interest because of their colocalization and concomitant allelic effect on plant height and other yield-related traits. LD-b_2H_7 was associated with PH, whereas LD-b_7H_58 was related to three traits (PH, GNSp and GWSp) that directly contributed to yield performance. Colocalization of root architecture loci with other traits is a well-known phenomenon (Khasanova et al., 2019; Danakumara et al., 2021). The allele effect detected in this study is much larger in LD blocks than in single markers, indicating the superiority of LD-based GWAS. In addition, when using single markers, the chances of missing data are higher as only one vector of effects can be observed. When using LD blocks, we can observe both types of effects on the studied traits.

The LD block LD-b_7H_58 could be an interesting target for crop improvement, as it contributes similarly to RB and yield increase. However, this block also results in an increase in plant height, which could contribute to lodging with all the unfavorable consequences for crop yield (Kuczyńska et al., 2013), but the increase in PH is relatively smaller compared with the mean value of MTAs detected in GWAS for this trait. Therefore, LD-b_7H_58 is presumed to be an important and promising target for future validations. Another interesting LD block (LD-b_2H_7) was located on the short arm of chromosome 2H. Near this region, the *PPD-H1* locus was identified as the major determinant of response to long photoperiods in barley (Turner et al., 2005). The *PPD-H1* locus encodes the *pseudo-response regulator* (*HvPRR37*) gene which is orthologous to the *Arabidopsis* gene *PRR7*. This gene is part of the plant circadian clock and its activity increases the expression of *VRN-H3*, the main promoter of flowering, when photoperiods increase above 12 h (Turner et al., 2005; Campoli et al., 2012). Turner et al. (2005) identified an SNP (G/T) at the *PPD-H1* locus, resulting in an amino acid change in the CCT domain, which is potentially responsible for the long photoperiod insensitivity. This has also been confirmed in a recent study (Sharma et al., 2020), suggesting that the region on the short arm of chromosome 2H plays an important role in shaping the root architecture. Many association-based studies with extensive germplasm collections have identified *PPD-H1* as mainly responsible for variations in flowering duration (Russell et al., 2016; He et al., 2019). In addition to the timing of flowering, pleiotropic effects of *PPD-H1* have been observed for many relevant agronomic and morphological traits, such as plant height, leaf size, root growth, and yield components (Ogrodowicz et al., 2017; Alqudah et al., 2018; Wiegmann et al., 2019; Ogrodowicz et al., 2020). *PPD-H1* appears to have site-specific effects on yield-related traits, especially (but not only) in association with earliness. The responsive/sensitive allele of *PPD-H1* has been associated with increased yield in sites where earliness is advantageous (e.g., early plants can escape drought at the end of the growing season). The yield effect can be explained by the pleiotropic effects of the responsive *PPD-H1* allele. This allele shortens the total growing period, prolongs the time of grain formation, and increases grain size. On the other hand, the nonresponsive *ppd-H1* allele has been shown to be associated with an increase in yield-related traits at sites where lateness is preferred to achieve higher yields (Wiegmann et al., 2019). However, the current long growing season which is characteristic of Northern Europe may increasingly change toward Mediterranean conditions as a result of climate change so that the ecological benefits of *PPD-H1* may disappear in some regions (Herzig et al., 2018). In their study, Jia et al. (2019) found that some QTL associated with root system depth and root spread angle traits were located near the *PPD-H1* genes. Root and shoot biomass showed high associations, including the functional SNP in *PPD-H1*, under osmotic stress and nonstress conditions (Valluru et al., 2017), which demonstrates the effect of *HvPPD-H1* on barley root growth as well. Arifuzzaman et al. (2017) also found that the head date gene *Vrn-H3* is significantly associated with shoot and root biomass; however, no associations were detected close to it in our GWAS panel. Even though the second promising LD block in the present study was indeed detected on the long arm of chromosome 7H, Vrn-H3 was found on the short arm of this chromosome. Recently, Voss-Fels et al. (2018) identified a context-specific interaction between flowering timing and root development, with early flowering lines with deeper root systems showing higher yield, especially under terminal drought conditions. These findings strongly prove the genetic link between roots and flowering time. Therefore, the regions associated with the abovementioned traits are important and promising targets for future validation in barley breeding.

### Marker translation effects on root biomass

In this study, the Ensembl Variant Effect Predictor (VEP) (McLaren et al., 2016) was used to show the effects of translation in selected SNPs on root biomass. The VEP analysis revealed that two SNP markers had a high translation effect, which had simultaneously positive significant effects on RB. SNP 1H_551859749 is located within LD-b_1H_4 and is annotated as a putative GABA transporter 2; it functions as a signaling molecule in plants (Li et al., 2021). Several studies have demonstrated the vital role of GABA (gamma-aminobutyric acid) in drought response of plants. During drought, GABA accumulation is a stress-specific response that helps regulate stomatal opening to prevent water loss (Mekonnen et al., 2016). Various responses to drought have been observed in different plant species, such as increased chlorophyll content, osmoregulation, antioxidant enzyme activity, enhancement of nitrogen recycling, protection of photosystem II, wax biosynthesis, fatty acid desaturase activity, and delay in leaf senescence (Rezaei-Chiyaneh et al., 2018; Hasan et al., 2021). The other SNP with a high marker translation effect was 2H_21581482 within the LD block on chromosome 2H (LD-b_2H_10). This marker was annotated as an aquaporin transporter. Aquaporins are membrane channels that belong to the superfamily of large intrinsic proteins. They play an essential role in maintaining cellular water content and osmotic homeostasis in plants under both control and water-deficient conditions. These proteins are also associated with the tolerance of plants to biotic and abiotic stresses (Li et al., 2015). Overexpression of aquaporins significantly improves the development of the root structure and leads to better tolerance to drought stress in plants such as wheat (Ayadi et al., 2019) and *Arabidopsis* (Patankar et al., 2019). Although some studies have reported the versatile roles of GABA and aquaporin transporters (Wang et al., 2020; Li et al., 2021), the associations of these genes with root development in spring barley remain elusive.

### Putative candidate genes for root-associated LD blocks

Eight genes harbored by the chosen LD blocks (LD-b_2H_7 and LD-b_7H_58) were discovered using selected annotation tools. The SNP markers within the block LD-b_2H_7 were associated with the following genes: HORVU2Hr1G007220, HORVU2Hr1G007340, HORVU2Hr1G007350, and HORVU2Hr1G008240. Wall-associated kinase-like 1 is a HORVU annotation associated with HORVU2Hr1G007340. No association was found for HORVU2Hr1G007220. Both HORVU2Hr1G007340 and HORVU2Hr1G007350 genes were associated with wall-associated kinase-like 1. Wall-associated kinases (WAKs) are receptors of the plasma membrane. They appear to be crosslinked with the cell wall material and can be detected in the cell wall by electron microscopy. WAKs are required for cell expansion and are involved in the response to pathogens. Their expression is activated by several environmental stimuli. According to the available data, WAKs play a role in cell expansion (Li et al., 2009). For example, they are required for cell elongation (Anderson et al., 2001; Wagner and Kohorn, 2001), which in turn is essential for root growth. Furthermore, in the present study, the direct association of HORVU with the flowering process was observed for the genes HORVU2Hr1G007340 and HORVU2Hr1G007350, both of which are associated with flowering promoting factor 1 (*FPF1*). To the best of our knowledge, this gene annotation has not been reported previously as the gene associated with both root growth and flower development in spring barley. This finding suggests that this region is associated with the previously mentioned spot on chromosome 2H that harbors flowering genes. *AtFPF1* in *Arabidopsis* encodes a small 12.6-kDa protein that regulates flowering and is involved in gibberellin signaling. It is expressed in apical meristems immediately after the photoperiodic induction of flowering. Genetic interactions of this gene with flowering time genes and floral organ identity suggest that it may be involved in modulating the competence to flower. *FPF1* is mainly expressed in roots and flowers and, to a lesser extent, in leaves. *OsRAA1*, a homolog of *FPF1* in rice, showed 58% similarity to *AtFPF1* at the amino acid level. Overexpression of *OsRAA1* induces pleiotropic phenotypes in transgenic rice plants, such as altered leaf shape, heading time, and root development (Kohorn et al., 2009). This observation suggests a possible interaction between the patterns of flowering time and root development. The last gene annotation (S-domain receptor-like protein kinase) for LD blocks discovered on chromosome 2H was registered for the gene HORVU2Hr1G008240. Receptor-like kinase (RLK) is a large protein family that includes more than 600 genes in *Arabidopsis* and 1100 genes in rice. A possible role in pollen development has been demonstrated for a type of the RLK gene (*LecRLK*). Disruption of the gene *LecRK-IV.2* leads to the formation of smaller pollens that remain stuck together and cannot be detached from the dehiscent anther, eventually resulting in male sterility (Smykal et al., 2004). In the present study, it is worth noting that the annotation and recognition of pollens were recorded for this gene. Although some level of relative expression of OsLecRK was found in both roots and plant tissues associated with anthers in rice (Peng et al., 2020), the functional associations of these genes with both root and pollen development have not been discovered previously in barley. During root growth and development, RLKs control many biological processes (Ou et al., 2021). Moreover, several RLKs have been identified as essential regulators of root hair development in *Arabidopsis* (Wei et al., 2018). The second LD-based block contained SNP markers associated with the following genes: HORVU7Hr1G099520, ENSRNA049488629, ENSRNA049488633, and HORVU7Hr1G101010. ENSRNA049488629/ENSRNA049488633 was annotated as a U2 spliceosomal RNA. In eukaryotic cells, pre-mRNA splicing is critical for processes such as expression of intron-containing genes, remodeling of protein–protein interaction networks, and regulation of transcript levels (Nilsen and Graveley, 2010). These important cellular functions are mediated by spliceosomes, which comprise five small nuclear ribonucleoproteins (nRNPs) and numerous non-nRNPs (Wahl et al., 2009). Plants are constantly exposed to various biotic and abiotic environmental stresses (Lamers et al., 2020). An interesting finding is that genes involved in stress responses are more likely to undergo an alternative splicing (AS) process (Laloum et al., 2018).

Therefore, AS in living plants may be a result of their adaptation to the new environment during land colonization (Mastrangelo et al., 2012). Thus, these annotations undeniably demonstrate the ability of plants to adapt to rapid changes in the internal and external environment, indicating that plasticity may be a defining feature of plant adaptation (Fritz et al., 2018). The next SNP markers within the LD block of interest were assigned to the following HORVU ID (HORVU7Hr1G099520 and HORVU7Hr1G101010) and were annotated, among others, as a serine–threonine/tyrosine–protein kinase/leucine-rich repeat domain superfamily. The annotation LRR receptor-like serine/threonine–protein kinase EFR was referred to as HORVU2Hr1G007220, indicating the possible links of this resistance gene with root development. Leucine-rich repeat receptor-like protein kinase (LRR-RLK) takes part in plant development and disease defense. In both plants and animals, cell surface receptors are involved in perceiving and processing external and internal signals that arrive at the cell surface. Many LRR-RLKs play an important role in both plant development and defense against pathogens because there is an overlap between these two pathways or because the same receptor recognizes multiple ligands (Oh et al., 2018). A gene that belongs to this gene family in *A. thaliana*—*GSO1* in coordination with *GSO2*—regulates root growth by controlling cell division and cell fate specification and controls seedling root growth by modulating sucrose response after germination (Racolta et al., 2014).

## Conclusions

In summary, the findings of our study offer new insights into the role of root traits in the yield performance of barley plants growing under natural field conditions where soil moisture varies day to day. The barley association panel analyzed in this study showed high variability in most of the agronomic traits. The results demonstrated the importance of the region on the short arm of chromosome 2H in the expression of root- and yield-related traits. Furthermore, the selected SNP markers with high translation effects showed great potential for further investigations of the adaptation of roots to drought. This study also highlighted the pleiotropic effect of the region with respect to heading time and other important agronomic traits, including root architecture. Because barley is widely considered as a model plant, the potential regions of interest identified in our study can be further validated to determine their application in barley breeding programs and to develop drought-tolerant genotypes in different species.

## Data availability statement

The original contributions presented in the study are included in the article/**Supplementary Material**. Further inquiries can be directed to the corresponding author.

## Author contributions

Conceptualization, PO, KM and AK. Methodology, PO and PK. Software, MM. Validation, PO, MM, PK and AK. Formal Analysis, MM and PK. Investigation, PO and MK. Resources, PO and KM. Data Curation, MM and PK. Writing – Original Draft Preparation, PO. Writing – Review and Editing, KM, MM, PK and AK. Visualization, MM. Supervision, AK. Project Administration, PO, KM and AK. Funding Acquisition, PO, KM and AK. All authors contributed to the article and approved the submitted version.
