# Supplementary material for: Genome-wide association study of agronomical and root-related traits in spring barley collection grown under field conditions
Source: Front Plant Sci. 2023 Jan 24;14:1077631. doi: 10.3389/fpls.2023.1077631 (PMC9902773; doi:10.3389/fpls.2023.1077631)
Supplement: Supplementary file 1 [file Image_1.pdf]

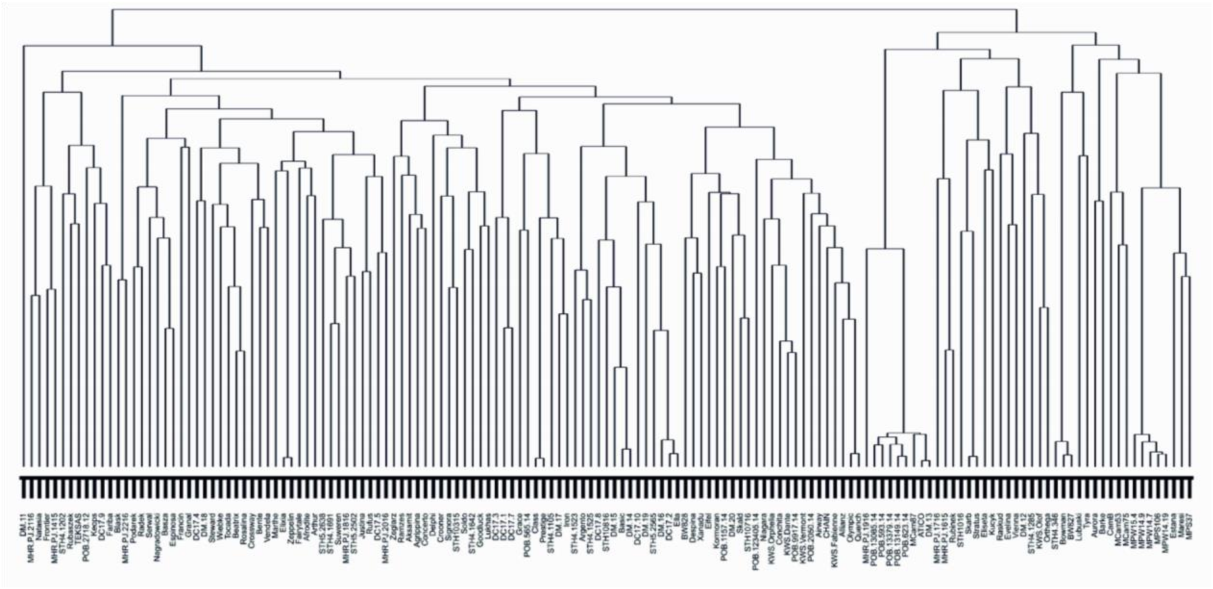

Supplementary Figure 1. Dendrogram of all 149 accessions constructed by hierarchical clustering using the average linkage method based on kinship matrix.
