# Supplementary material for: Genome-wide association study of agronomical and root-related traits in spring barley collection grown under field conditions
Source: Front Plant Sci. 2023 Jan 24;14:1077631. doi: 10.3389/fpls.2023.1077631 (PMC9902773; doi:10.3389/fpls.2023.1077631)
Supplement: Supplementary file 3 [file Table_2.docx]

Supplementary Table 2. The detailed description of the fertilization used in the field experiments

| Fertilization | Dose |
| --- | --- |
| Granular fertilizer NPK (MgS) 5-15-30-(2-7) (POLIFOSKA) | 600 kg per hectare |
| Granular fertilizer ammonium sulphate | 12,5 kg per hectare |
| Foliar fertilizer Wuxal AminoPlus | 5 l per hectare ha |
| Herbicide Granstar Ultra SX 50 SG | 40 g per hectare |
| Fungicide Osiris | 2 l per hectare |
