# Supplementary material for: Genome-wide association study of agronomical and root-related traits in spring barley collection grown under field conditions
Source: Front Plant Sci. 2023 Jan 24;14:1077631. doi: 10.3389/fpls.2023.1077631 (PMC9902773; doi:10.3389/fpls.2023.1077631)
Supplement: Supplementary file 4 [file Table_3.docx]

Supplementary Table 3. Characteristics of root- and yield-related traits observed in the experiment.

| Trait name | 2017 | | | | 2018 | | | | 2020 | | | |
| --- | --- | --- | --- | --- | --- | --- | --- | --- | --- | --- | --- | --- |
|  | Min | Max | Mean | Std error | Min | Max | Mean | Std error | Min | Max | Mean | Std error |
| Total biomass (g) | 2.49 | 18.57 | 10.11 | 0.25 | 9.38 | 32.10 | 18.83 | 0.39 | 5.30 | 18.43 | 10.19 | 0.19 |
| Root biomass (g) | 0.07 | 0.86 | 0.41 | 0.01 | 0.38 | 4.44 | 1.81 | 0.06 | 0.45 | 3.03 | 1.56 | 0.05 |
| Root depth (cm) | 10.00 | 23.00 | 16.09 | 0.19 | 7.00 | 16.33 | 11.89 | 0.13 | 10.33 | 24.67 | 16.24 | 0.22 |
| Number of productive tillers | 1.67 | 9.33 | 4.78 | 0.13 | 4.67 | 17.00 | 9.02 | 0.17 | 3.00 | 8.33 | 5.21 | 0.09 |
| Total number of tillers | 2.00 | 9.67 | 5.29 | 0.13 | 5.33 | 18.00 | 10.12 | 0.18 | 3.33 | 8.67 | 5.43 | 0.09 |
| Plant height (cm) | 41.33 | 62.33 | 51.86 | 0.35 | 42.67 | 63.00 | 54.38 | 0.32 | 39.33 | 65.67 | 51.78 | 0.44 |
| Spike length (cm) | 5.43 | 10.20 | 7.98 | 0.08 | 5.00 | 9.10 | 6.51 | 0.05 | 5.33 | 9.17 | 6.85 | 0.06 |
| Number of grains per spike | 12.57 | 25.90 | 18.82 | 0.19 | 9.00 | 22.37 | 17.21 | 0.18 | 12.83 | 23.17 | 17.61 | 0.15 |
| Weight of grains per spike (g) | 0.51 | 1.38 | 0.88 | 0.01 | 0.34 | 1.20 | 0.80 | 0.01 | 0.51 | 1.17 | 0.83 | 0.01 |
| Weight of grains per plant (g) | 1.16 | 7.76 | 4.10 | 0.11 | 2.28 | 13.08 | 7.16 | 0.16 | 2.18 | 7.18 | 4.30 | 0.08 |
| Thousand-grain weight (g) | 31.86 | 56.75 | 46.53 | 0.37 | 32.72 | 53.88 | 46.01 | 0.32 | 35.22 | 54.22 | 47.17 | 0.29 |
| Electrical capacitance I | −1.44 | 3.21 | 0.01 | 0.07 | −1.40 | 2.18 | 0.00 | 0.05 | −1.37 | 1.95 | −0.01 | 0.05 |
| Electrical capacitance II | −1.73 | 1.75 | 0.01 | 0.06 | −1.41 | 1.82 | 0.00 | 0.05 | −1.50 | 2.41 | −0.01 | 0.06 |
| Electrical capacitance III | −1.42 | 2.72 | 0.01 | 0.06 | −1.53 | 1.82 | 0.00 | 0.05 | −1.42 | 2.66 | 0.01 | 0.06 |
