# Supplementary material for: Genome-wide association study of agronomical and root-related traits in spring barley collection grown under field conditions
Source: Front Plant Sci. 2023 Jan 24;14:1077631. doi: 10.3389/fpls.2023.1077631 (PMC9902773; doi:10.3389/fpls.2023.1077631)
Supplement: Supplementary file 6 [file Table_5.docx]

Supplementary Table 5. ANOVA results and heritability for studied traits.

| Trait | *P* value for years | G variance | G s.e. | G × Y variance | G × Y s.e. | Error variance | Error s.e. | Heritability | G var/s.e. | G × Y var/s.e. |
| --- | --- | --- | --- | --- | --- | --- | --- | --- | --- | --- |
| Total biomass (g) | <0.001 | 1.944 | 0.688 | 4.089 | 0.900 | 18.672 | 0.884 | 36.059 | 2.826 | 4.540 |
| Root biomass (g) | <0.001 | 0.007 | 0.014 | 0.187 | 0.024 | 0.303 | 0.014 | 7.028 | 0.506 | 7.738 |
| Root depth (cm) | <0.001 | 0.236 | 0.245 | 2.552 | 0.398 | 6.398 | 0.302 | 13.093 | 0.960 | 6.405 |
| Number of productive tillers | <0.001 | 0.476 | 0.154 | 0.672 | 0.197 | 4.648 | 0.220 | 39.070 | 3.082 | 3.410 |
| Total number of tillers | <0.001 | 0.419 | 0.156 | 0.863 | 0.210 | 4.579 | 0.217 | 34.408 | 2.681 | 4.111 |
| Plant height (cm) | <0.001 | 7.055 | 1.405 | 6.704 | 1.178 | 21.093 | 0.997 | 60.594 | 5.019 | 5.693 |
| Spike length (cm) | <0.001 | 0.178 | 0.042 | 0.230 | 0.041 | 0.752 | 0.036 | 52.632 | 4.294 | 5.555 |
| Number of grains per spike | <0.001 | 0.932 | 0.263 | 1.517 | 0.305 | 6.009 | 0.284 | 44.230 | 3.541 | 4.978 |
| Weight of grains per spike (g) | <0.001 | 0.004 | 0.001 | 0.008 | 0.001 | 0.021 | 0.001 | 42.285 | 3.367 | 6.112 |
| Weight of grains per plant (g) | <0.001 | 0.372 | 0.127 | 0.673 | 0.165 | 3.591 | 0.170 | 37.277 | 2.931 | 4.092 |
| Thousand-grain weight (g) | 0.017 | 4.120 | 1.009 | 9.219 | 1.013 | 8.892 | 0.420 | 50.301 | 4.082 | 9.104 |
| Electrical capacitance I | – | 0.066 | 0.026 | 0.145 | 0.036 | 0.785 | 0.037 | 32.795 | 2.550 | 4.057 |
| Electrical capacitance II | – |  | 0.020 | 0.147 | 0.038 | 0.854 | 0.040 |  |  | 3.861 |
| Electrical capacitance III | – | 0.034 | 0.024 | 0.161 | 0.038 | 0.804 | 0.038 | 19.287 | 1.442 | 4.295 |

G—genotypes, Y—years, s.e.—standard error of variance component
