# Supplementary material for: Genome-wide association study of agronomical and root-related traits in spring barley collection grown under field conditions
Source: Front Plant Sci. 2023 Jan 24;14:1077631. doi: 10.3389/fpls.2023.1077631 (PMC9902773; doi:10.3389/fpls.2023.1077631)
Supplement: Supplementary file 9 [file Table_8.docx]

Supplementary Table 8. Summary of LD blocks in 149 barley genotypes.

| Chr. | No. of blocks | SNPs per block | |
| --- | --- | --- | --- |
|  |  | Avg | Max |
| 1H | 5 | 9.8 | 32 |
| 2H | 15 | 4.9 | 13 |
| 3H | 3 | 5.7 | 9 |
| 4H | 11 | 4.8 | 10 |
| 5H | 8 | 4.1 | 9 |
| 6H | 8 | 7.9 | 22 |
| 7H | 8 | 5.8 | 16 |
| Un | 1 | 3.0 | 3.0 |
